# Prevention and early intervention screening for inherited ocular diseases in Saudi Arabia: a national perspective

**DOI:** 10.3389/fopht.2025.1660483

**Published:** 2026-01-06

**Authors:** Mariam M. AlEissa, Malak Abedalthagafi, Deepak P. Edward

**Affiliations:** 1 Department of Research, King Khaled Eye Specialist Hospital (KKESH) Research Center, Riyadh, Saudi Arabia; 2 College of Medicine, AlFaisal University, Riyadh, Saudi Arabia; 3 Public Health Observatory, Ministry of Health, Riyadh, Saudi Arabia; 4 Public Health Authority, Public Health Lab, Molecular Genetics Department, Riyadh, Saudi Arabia; 5 Computational Sciences Department at the Centre for Genomic Medicine (CGM), King Faisal Specialist Hospital & Research Center, Riyadh, Saudi Arabia; 6 Department of Pathology and Laboratory Medicine, Tufts University School of Medicine, Boston, MA, United States

**Keywords:** PCG, ocular diseases, vision impairment, genetic screening, AI, Telemedicine, prevention

## Abstract

Consanguineous marriages are common, particularly in Saudi Arabia, where approximately half of all marriages are consanguineous. The likelihood of autosomal recessive (AR) genetic abnormalities, especially rare diseases (RDs) that present long-term health issues, is significantly increased by this trend, making Inherited Ocular Diseases (IODs) a public health concern in Saudi Arabia. The common IODs include Primary Congenital Glaucoma (PCG), Retinitis Pigmentosa (RP), and Leber Congenital Amaurosis (LCA). To date, there are no national initiatives for screening programs to detect, prevent, and manage IOD. This review aims to evaluate the public health landscape of IOD in Saudi Arabia, including epidemiology and genetic factors. It highlights the need for a national framework to facilitate screening, prevention, and early intervention. Cost-effectiveness evaluation, early genetic screening, and counseling can drastically lower the long-term medical expenses related to IOD management. Outreach programs can be used to address issues, including cultural acceptance and equitable access to screening services, which are needed. Further carrier screening for at-risk families accompanied by genetic counseling decreases new IOD cases and provides better family planning for impacted populations. These are among the expected results, which will eventually enhance health outcomes and ensure the long-term viability of Saudi Arabia’s healthcare system. In alignment with Vision 2030’s futuristic pipeline for screening, introducing telemedicine and AI-driven predictive tools can enhance accessibility and precision in screening programs.

## 1 Introduction

Consanguineous marriages (CMs) are common in the Middle East and North Africa (MENA) region. While prevalence rates vary across different countries, this widely encouraged practice is often associated with economic factors and the reinforcement of social ties (1). In Saudi Arabia, nearly 50% of the population engages in CM, with half of these marriages occurring between first cousins (2). Socioeconomic and demographic factors significantly influence marriage decisions, increasing the risk of autosomal recessive (AR) genetic disorders (1, 2). Rare diseases (RDs) often require long-term care or rehabilitation to maintain a normal quality of life. These conditions lead to chronic health issues that impose financial and emotional burdens on patients, their families, and healthcare providers (3, 4). However, some diseases can be prevented before birth or mitigated through early screening, reducing their devastating impact (5).

Inherited Ocular Diseases (IODs) encompass a broad spectrum of hereditary disorders that impair vision, ranging from developmental anomalies such as retinal dystrophies, congenital cataracts, and microphthalmia to hereditary optic neuropathies and anophthalmia. Among these, retinal dystrophies like Leber Congenital Amaurosis (LCA) and Retinitis Pigmentosa (RP) affect approximately 1 in 1,000 people worldwide, often leading to early-onset blindness or severe visual impairment (6). Although IODs are typically monogenic disorders, complex inheritance patterns may be involved in some instances (6). Genetic testing has proven highly effective in enabling personalized treatment and early intervention for hereditary conditions (7). Additionally, artificial intelligence (AI) and machine learning (ML) can enhance early diagnosis and identify high-risk individuals, allowing timely surgical interventions that significantly improve visual outcomes (8). For example, early detection of congenital cataracts facilitates timely surgical intervention, thereby greatly enhancing the visual prognosis (9). Identifying genetic risks early supports informed decision-making for prospective parents and family planning. Proactively managing genetic disorders can substantially reduce the long-term economic burden on healthcare systems. Genetic testing plays a crucial role in the management of inherited diseases and reproductive planning (10). Studies have shown that countries implementing genetic screening programs experience lower healthcare costs due to early intervention and management, thereby reducing the long-term care required for inherited disorders (11). However, a significant barrier to widespread genetic testing is the lack of public awareness and limited accessibility to genetic screening services (12).

## 2 Genetic landscape of inherited ocular diseases in Arabs

A comprehensive analysis across various Arab countries has highlighted the distribution of different Inherited Retinal Dystrophies (IRDs). For example, in Tunisia, the most prevalent IRDs include rod-cone dystrophies (19%), Usher syndrome (19%), Bardet-Biedl syndrome (17%), and achromatopsia (8%). The most frequently mutated genes in the Tunisian population include *ABCA4* (9%), *RPE65* (9%), and *GPR98* (6.3%) (13, 14). These findings underscore the substantial burden of inherited ocular diseases (IODs) in the Middle East, and the need for early diagnosis, targeted genetic screening, and personalized healthcare strategies to prevent, manage, and reduce the incidence of these conditions in the region (6).

With the increasing prevalence of genetic disorders in Saudi Arabia, IODs pose a significant health challenge (14). Retinal diseases such as Stargardt disease, Leber congenital amaurosis (LCA), retinitis pigmentosa (RP), and cone-rod dystrophies are more commonly observed. Mutations in specific genes, including *RHO, USH2A*, and *RPGR*, have been strongly linked to RP, increasing its prevalence in the Saudi population (15). Additionally, IODs contribute significantly to the prevalence of anterior segment diseases such as congenital glaucoma, keratoconus, and corneal dystrophies (macular and lattice) in Saudi Arabia. Furthermore, mutations in the *CYP1B1* gene are strongly associated with congenital glaucoma. High consanguinity rates in Saudi Arabia contribute to an increased prevalence of IOD and high genomic homozygosity of pathogenic founder variants (16).

The presence of founder mutations and high levels of homozygosity have been noted in genes such as *TULP1, ABCA4*, and *RPGRIP1*, with studies showing that 93% of affected individuals carry homozygous mutations (17) (13). This genetic heterogeneity indicates the presence of multiple pathogenic variants across various IRDs, including RP and LCA (17, 18). These findings highlight the importance of genetic screening, early intervention, and genetic counseling. Identifying the genetic components of these diseases will facilitate further research, paving the way for precision medicine and future tailored treatments (19).

For instance, keratoconus is more prevalent in the Middle East due to a combination of genetic predisposition and environmental factors, such as exposure to ultraviolet light and eye-rubbing habits (20).

## 3 Epidemiology and burden of inherited ocular diseases in Saudi Arabia

Ocular genetic disorders are notably prevalent in Saudi Arabia and the broader Middle East, primarily due to high consanguinity rates and population-specific genetic mutations (21). Supported by data from the literature, this section provides an overview of the burden of these disorders in Saudi Arabia and the region.

### 3.2 Primary congenital glaucoma and genetic screening in Saudi Arabia

Primary Congenital Glaucoma (PCG) is a rare form of glaucoma that typically presents within the first few years of life with symptoms such as photophobia, excessive tearing, and corneal clouding (22). Abnormal development of the anterior chamber angle or trabeculodysgenesis impairs aqueous outflow by affecting the trabecular meshwork and Schlemm’s canal (Badawi et al., 2019). This leads to elevated intraocular pressure, resulting in corneal edema, Haab striae, buphthalmos, and early optic nerve cupping if left untreated (Abu-Amero et al., 2014; Kaur et al., 2011). Due to its severe phenotype, PCG often requires multiple surgical interventions and lifelong medical care, contributing to a substantial socioeconomic burden (23). The incidence of PCG varies globally, with a frequency of 1 in 10,000 to 20,000 live births worldwide (24). However, in Saudi Arabia, relying on incidence-based data, incidence is significantly higher, estimated at 1 in 2,500 to 3,000 live births, translating to approximately 100 new cases annually (5). Widely recognized as an autosomal recessive disorder, mutations in the *CYP1B1* gene disrupt normal ocular development and affect the enzyme responsible for metabolizing endogenous chemicals in the eye (23). Studies conducted in Saudi Arabia have consistently linked *CYP1B1* mutations to PCG, emphasizing the need for targeted genetic screening programs (25).

A study published by Aleissa et al. (2022) analyzed the most frequently carried pathogenic alleles in the Saudi population by comparing three genetic databases. The study reported the minor allele frequency of the *CYP1B1* gene variant (rs28936700, c.182G>A), which is associated with glaucoma. High allele frequency rates were found in two Saudi databases, specifically 2.51% in the Saudi Human Genome Project database (dbSNP) and 1.31% in the King Abdullah International Medical Research Center Genomic Database (KGD) (16). In contrast, the variant’s global allele frequency in the Genome Aggregation Database (gnomAD) was reported as 0.03%. To address this issue, a national screening program has been proposed for implementation in the coming years to facilitate early detection and intervention (26).

#### 3.2.1 Inherited retinal diseases

##### 3.2.1.1 Saudi Arabia

A hospital-based genetic registry study involving 650 patients identified retinitis pigmentosa (40%), cone-rod dystrophy (14%), Leber congenital amaurosis (11%), and Bardet-Biedl syndrome (8%) as the most common IRDs. The study reported a 37.6% consanguinity rate, with autosomal recessive (AR) inheritance observed in 78% of cases. Genetic testing successfully identified pathogenic or likely pathogenic mutations in 80% of the tested individuals (18, 29) in the United Arab Emirates (UAE):

In a cohort of 74 families, genetic testing achieved a high diagnostic yield of 90.3%, identifying 69 disease-causing variants across 40 genes. The most frequently diagnosed conditions were rod-cone dystrophies (22%), Stargardt disease (18%), and cone-rod dystrophies (13%). Notably, ABCA4 gene mutations accounted for 24.8% of cases (27).

#### 3.2.2 Keratoconus

##### 3.2.1.2 Saudi Arabia, Middle East/North Africa

Keratoconus is more prevalent in Saudi Arabia and the Middle East compared to Western populations. For instance, studies have reported prevalence rates of approximately 3,330 per 100,000 in Lebanon and 2,500 per 100,000 in Iran, which are significantly higher than the estimated 50 per 100,000 among Caucasians (28–31). Family history is a notable risk factor for keratoconus, with reported rates of 16% in Saudi Arabia and 15% in Iran. This higher prevalence has been linked to cultural and religious practices, particularly consanguineous marriages, which contribute to the genetic predisposition for the disease (28–31). The wide range of climate and environmental variations influenced by regional factors, particularly the high-UV-exposure latitudes found in Saudi Arabia and neighboring Gulf countries, may contribute to the higher incidence of keratoconus (Mohamed, 2025). Elements such as dust, dryness, and sun exposure may also contribute to disease development (32). Additional lifestyle factors, including limited use of protective eyewear, frequent eye rubbing, and exposure to pollution, further increase the risk (33, 34). The high prevalence of allergic conjunctivitis, particularly vernal keratoconjunctivitis in the Middle East, also contributes to keratoconus (35).

#### 3.2.3 The multifaceted burden of IODS

Multifaceted burdens imposed by IOD reflected on the patients, families, and society, encompassing psychological, health, and economic challenges:

##### 3.2.3.1 The burden on individuals

###### 3.2.3.1.1 Health and vision impairment

Inherited retinal diseases (IRDs) can lead to severe vision loss or blindness, significantly affecting daily activities and quality of life.

###### 3.2.3.1.2 Psychological impact

Vision impairment from IRDs often manifests in childhood, leading to lifelong challenges. Individuals may experience reduced quality of life due to the progressive nature of these diseases (25).

##### 3.2.3.2 The burden on families

###### 3.2.3.2.1 Emotional and social strain

Families often bear the primary responsibility for care, which can lead to emotional distress and social challenges. A research study highlighted that living with an IRD affects the financial stability of the individual and their family.

###### 3.2.3.2.2 Caregiving responsibilities

Family members often become primary caregivers, balancing work and caregiving duties, which can increase stress and impact family dynamics (16).

###### 3.2.3.2.3 Psychosocial burden

The Quality of life varies across IOD, affecting mobility with retinal dystrophies, early-onset visual disability in the case of congenital glaucoma, and educational performance affected by impairment of central vision in optic nerve disorders, each of which diseases associated with psychosocial burdens affecting the patients and families (9, 22, 36).

##### 3.2.3.3 Burden on society

The costs to society for inherited ocular disorders are difficult to compute, as specific data in many areas is lacking and would depend on the severity of disability and other variables, but are likely significant (16). However, costs can be calculated under the broad categories shown in **Table 1** (Supplementary). These include direct medical costs, direct non-medical costs, indirect costs, and intangible costs.

**Table 1:** Presents a breakdown of the stages of the treatment in IOD patients, which require specific costs for each stage (38, 56, 90, 91).

Spending	Justification	Specific costs
Direct Medical Costs	These expenses are related to healthcare services provided to manage the condition.	Diagnostics and Testing: Genetic testing, molecular diagnostics, and imaging (e.g., OCT, fundus photography). Treatments: Medical interventions include anti-VEGF injections, gene therapy (e.g., Luxturna), or other innovative treatments. Surgical Procedures: Surgeries such as retinal detachment repair or cataract removal. Follow-up and Monitoring: Regular specialist consultations and monitoring of disease progression.
Direct Non-Medical Costs	These costs are associated with services and adjustments that support daily living:	Assistive Devices: Costs of low-vision aids, magnifiers, braille materials, and screen readers. Transportation: Travel expenses for hospital visits, particularly for individuals residing in remote areas. Home Modifications: Alterations to homes, such as improved lighting or tactile guidance systems, that support individuals with low vision.
Indirect Costs	These costs reflect the economic impact of productivity loss and caregiving.	Loss of Productivity: Reduced ability to work or early retirement due to visual impairment. Caregiver Costs: Lost income and time for family members who act as caregivers. Educational Impact: The Costs of specialized education or vocational training for children with vision impairment.
Intangible Costs	These are non-monetary costs related to quality of life and emotional well-being.	Psychological Impact: Emotional distress, depression, and anxiety are experienced by patients and families. Social Isolation: Reduced participation in social and community activities. Stigma and Discrimination: Challenges in societal acceptance and integration.

##### 3.2.3.4 Economic burden

The economic burden of IRDs is substantial. In the United States, the costs associated with IRDs accounted for over 60% of total healthcare costs, highlighting their significant impact on healthcare systems (25). On the other hand, screening interventions have substantially lower costs (37). Vision loss from IRDs often manifests in childhood, meaning some individuals live with vision impairment for their entire lives, leading to reduced productivity and increased reliance on social support systems.

Due to a knowledge gap in studies on the cost of illness in KSA, it is challenging to estimate the average cost per patient. However, average annual costs were reported in Singapore and Canada at $6,926 and $275,045, respectively (38). World Bank data shows that healthcare spending per capita in KSA is about 1.5 times higher than in Singapore. Applying this ratio to SA, the annual expenditure per patient would be $10,389 (34). PCG patients may incur costs exceeding $10,000 due to the need for multiple surgeries (27).

There is an urgent need for a national screening program for inherited ocular disorders in a population like Saudi Arabia, with a high prevalence of inherited ocular disease due to consanguineous marriages. Screening for specific genetic diseases like inherited cancers, Epidermolysis Bullosa, PCG, and other ocular diseases would benefit in many ways (39–43). Notwithstanding premarital screening tests (PMSTs) and newborn screening (NBS) in Saudi Arabia, which still cover only a few diseases (44, 45). One way is early disease detection during screening, along with early interventions to prevent disease progression and mutation-focused molecular therapy. Early medical and surgical treatments for certain diseases, such as cataracts and glaucoma, can prevent irreversible vision loss (46). In addition, detecting cases with specific mutations and, like any other rare disease, introducing cascade screening (47), further genetic counseling assists at-risk families in understanding their genetic risks, making informed decisions about treatment and prevention, reproductive options, and family planning, implementing lifestyle modifications (48, 49). High prevalence of consanguinity in Saudi Arabia increases the rate of AR disorders like IODs, justifying an early genetics screening and counseling program (50, 51).

The existing healthcare infrastructure in South Africa is transforming to integrate prevention into primary care for non-communicable diseases (52). The current screening program in SA focuses on preventing the high prevalence of hematological diseases through premarital screening, providing early education and screening (53). Furthermore, the newborn screening program is limited to endocrine and metabolic diseases (45). The introduction of a new screening model targeting families with rare diseases and providing them with targeted carrier screening will prevent future cases within affected families (47). The limitation would be identifying and targeting those families in rural areas where they are less likely to utilize preventive services (53).

The efficiency and cost-effectiveness of the screening program have increased due to recent developments in genetic testing technologies, such as next-generation sequencing (NGS), which enables the simultaneous screening of multiple genetic variants (54).

### 3.3 Future directions and Saudi vision 2030

The framework of Vision 2030 is to prioritize precision healthcare through prevention and early intervention, activating digital-health integration and AI-driven screening, to reduce disease burden in Saudi Arabia (55, 56). The role of genetic screening in managing IODs is crucial, particularly in Saudi Arabia, where healthcare policies are increasingly focused on early intervention and prevention strategies. The proposed nationwide genetic screening program aligns with Saudi Vision 2030, which emphasizes preventive healthcare initiatives to reduce the burden of inherited disorders (56).

### 3.4 Design and implementation of programs

The United States delivers a range of carrier screening programs based on guidelines and resources. Those programs effectively reduce the rate of new cases and provide an early intervention for those who need it (57). In Europe, the United Kingdom and Germany established a cost-effective system to improve health outcomes, where personalized screening starts with genetic counseling (57). For the prevention of IOD, an action plan needs to be implemented by primarily identifying families at risk, which requires integrating EHR, newborn, and premarital screening data, as well as national databases and primary-care networks in high-consanguinity regions. Further activate mobile screening units and tele-ophthalmology to benefit the population coverage, bridge gaps in rural areas where access is limited (58–60).

A structured design is necessary for IOD-related screening programs. Taking PCG as an example, a pilot screening program should be implemented at the national level. Families with a documented history of PCG and those identified as possible carriers will be the pilot’s focus. Hereditary testing for CYP1B1 mutations, a recognized hereditary cause of PCG, will be part of the screening process (22). Genetic counseling will be given to at-risk individuals after they have been identified to address the consequences, reproductive hazards, and possible preventative measures (58).

### 3.5 Ethical and social considerations

The multifaceted ethical implications of genetic screening necessitate a balance between individual rights and public health goals, encompassing cultural sensitivity, avoidance of stigmatization of the patient or their family, informed consent, and data privacy. The confidentiality of personal and family history is maintained by obtaining adequate informed consent before genetic screening, ensuring that the participant is aware of the implications of the test on themselves or their family (61, 62). Disclosing sensitive data regarding genetic risk might be associated with ethical dilemmas, where it may raise pressure and anxiety, especially when it is related to reproductive choices (63, 64).

Genetic screening for IODs presents significant ethical challenges, particularly in populations with high rates of consanguinity. It is imperative to balance individual rights with public health benefits by addressing the following:

#### 3.5.1 Data privacy and informed consent

**Figure 1:**
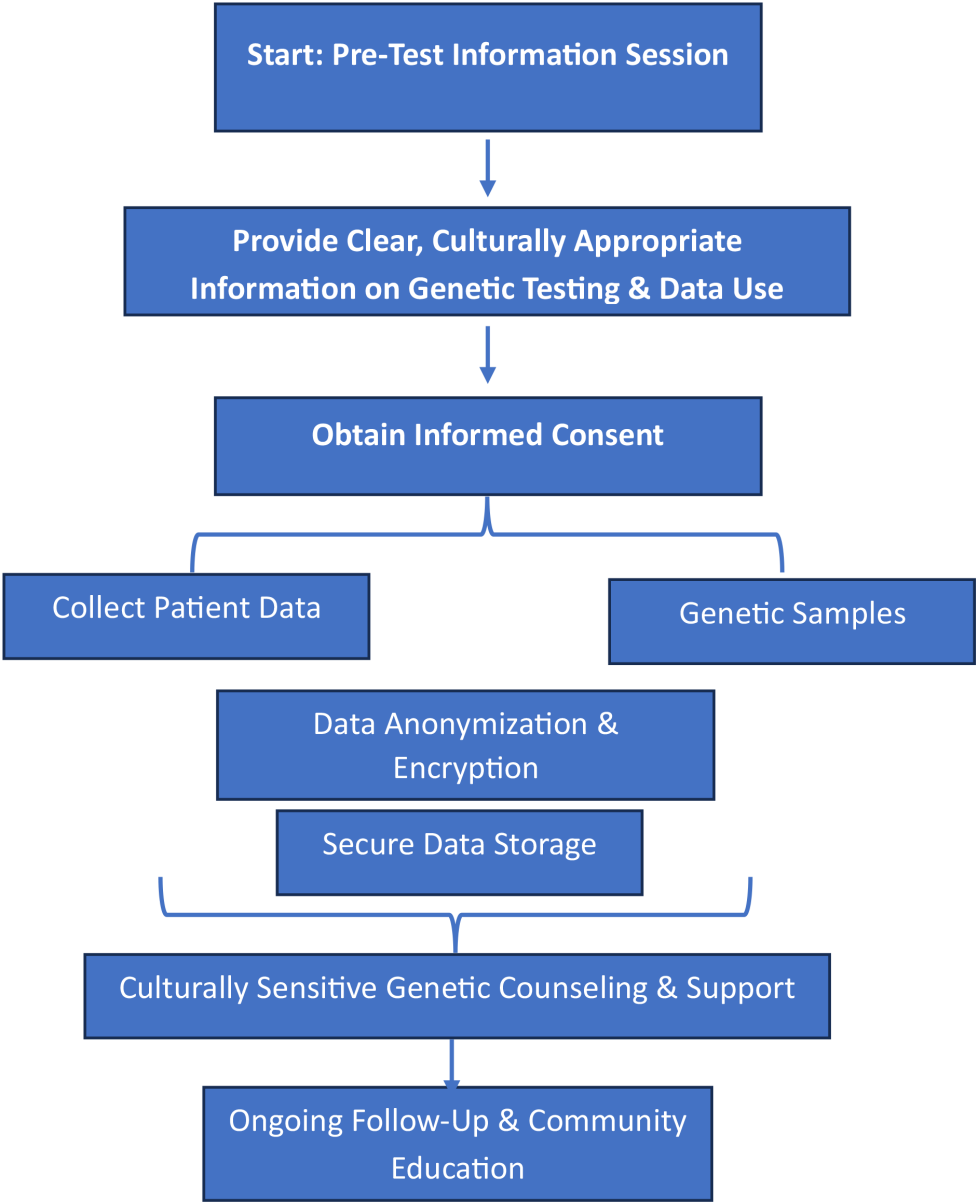
Ethical framework flow diagram. This flow diagram outlines the ethical processes involved in genetic screening for inherited ocular diseases, from obtaining informed consent to providing ongoing support. It emphasizes data privacy, culturally sensitive counseling, and continuous follow-up (59, 65).

A multi-step informed consent process must be in place. Before testing, participants should be provided with clear, culturally appropriate information on the implications of genetic screening, the potential for incidental findings, and the measures taken to protect their data (59, 65). Data Security Measures: All genetic data should be anonymized and stored using state-of-the-art encryption protocols. A dedicated flowchart (see **Figure 1**) can outline how consent is obtained, data is anonymized, and secure access is maintained (59, 65).

#### 3.5.2 Cultural sensitivity and stigmatization

Culturally Tailored Counseling: Genetic counseling services should be provided by culturally competent practitioners who understand local customs and sensitivities. This approach minimizes the risk of stigmatization and helps families make informed decisions (59, 65). Public Education Initiatives: Outreach programs should employ culturally resonant messaging to educate communities about the benefits of early screening and carrier testing (59, 65).

#### 3.5.3 Balancing individual and public health interests

Ethical Dilemmas: Disclosing genetic risk can cause psychological distress. Ethical protocols must be established to offer psychological support and ensure that individuals’ decisions whether to participate in screening or not are fully respected (59, 65).

#### 3.5.4 Cultural considerations

Although genetic screening is common in Saudi Arabia, cultural and religious beliefs may have an impact on some families’ decisions to participate. The program must be approached with cultural sensitivity and provide families with comprehensive information and support, enabling them to make informed decisions (58). Another issue is equitable access to genetic screening, especially for impoverished and rural communities with limited access to healthcare. Outreach initiatives and mobile genetic testing units can be utilized to address this issue and ensure broader nationwide coverage and participation (66, 67).

Equitable Access: Special attention is needed to ensure that rural and underserved populations are not excluded. Mobile testing units and telemedicine initiatives can help bridge the accessibility gap.

## 4 Clinical examination/screening

Clinical examination remains a cornerstone of ocular diagnosis. Tools like slit-lamp biomicroscopy, fundoscopy, and advanced imaging technologies, such as optical coherence tomography (OCT), allow for detailed evaluation of ocular structures and functional assessments (61). These methods are particularly effective for diagnosing conditions with distinctive clinical features, such as many inherited ocular disorders, and can provide immediate insights during a single visit. Functional tests, such as visual field analysis or electroretinography, are valuable for assessing the extent of visual impairment and guiding treatment plans (69). However, clinical examination/screening for inherited ocular disorders has limitations. Phenotypic overlap among different inherited disorders can lead to diagnostic uncertainty, and many conditions are not detectable until significant structural or functional changes occur (70). Furthermore, variability in clinical expertise may lead to inconsistencies in diagnosis and treatment.

## 5 Genetic Screening

Genetic screening offers a molecular-level understanding of ocular disorders, enabling early and precise diagnosis. This approach identifies specific mutations responsible for diseases, often before clinical symptoms manifest, and provides an accurate diagnosis when clinical findings are ambiguous or overlap with those of other disorders (36). For instance, genetic screening has been transformative in diagnosing inherited retinal diseases (IRDs), allowing for the identification of subtypes critical for gene-specific therapies, such as Voretigene neparvovec (Luxturna) for RPE65-associated retinal dystrophy (70). Additionally, genetic testing provides insight into inheritance patterns, allowing for family risk assessments and informed reproductive planning (71).

Despite its advantages, genetic screening is not without challenges. High costs and limited accessibility in resource-constrained settings pose significant barriers to the effective delivery of healthcare. Furthermore, the interpretation of genetic findings requires specialized expertise, and some results may be inconclusive due to unknown or poorly understood variants (36, 68). Both clinical examination and genetic screening are indispensable in the diagnosis of ocular disorders. Clinical examination excels in real-time functional and structural evaluation, while genetic screening provides molecular precision and prognostic insights. Together, these methods enable comprehensive care from early diagnosis to targeted treatment and genetic counseling, underscoring the importance of integrating both approaches into clinical practice (71, 72).

### 5.1 Strategies for prevention and screening of IOD

- Public campaigns raise awareness about IOD, highlighting the value of screening for carriers or undiagnosed cases (73).
- Creating a national screening program that focuses on high-risk individuals who have an affected family member and look for undiagnosed cases or potential carriers (73, 74), introducing new innovative technologies for genetics or clinical screening, and implementing them in the health care system (74).
- The value of expanding genetics counseling services lies in providing families affected by IOD with trained counselors who offer personalized care based on family history and tailored screening tests for each family, thereby improving patient outcomes. Further, facilitate decision-making and future family planning (75).
- Sharing resources will improve overall healthcare delivery. Expanding the partnership with national and international organizations will support resource allocation and enhance funding recruitment, facilitating the development of new therapies and management methods (76).
- With the advancement of the present technologies, which assess the improvement of healthcare delivery, starting with genetics testing for the identification of IOD and personalized treatment plans, with the advancement of gene therapy and gene-editing tools (77).
- Introducing remote screening (Telemedicine)or Tele-Ophthalmology for remote screening and consultations in areas where it is difficult to find eye care, utilizing wearable devices providing real-time monitoring of eye health.
- Providing a predictive AI and Machine learning (ML) modelling to detect high risk, identify disease progression, and enhance decision-making (77–79).

### 5.2 Screening procedures

**Figure 2:**
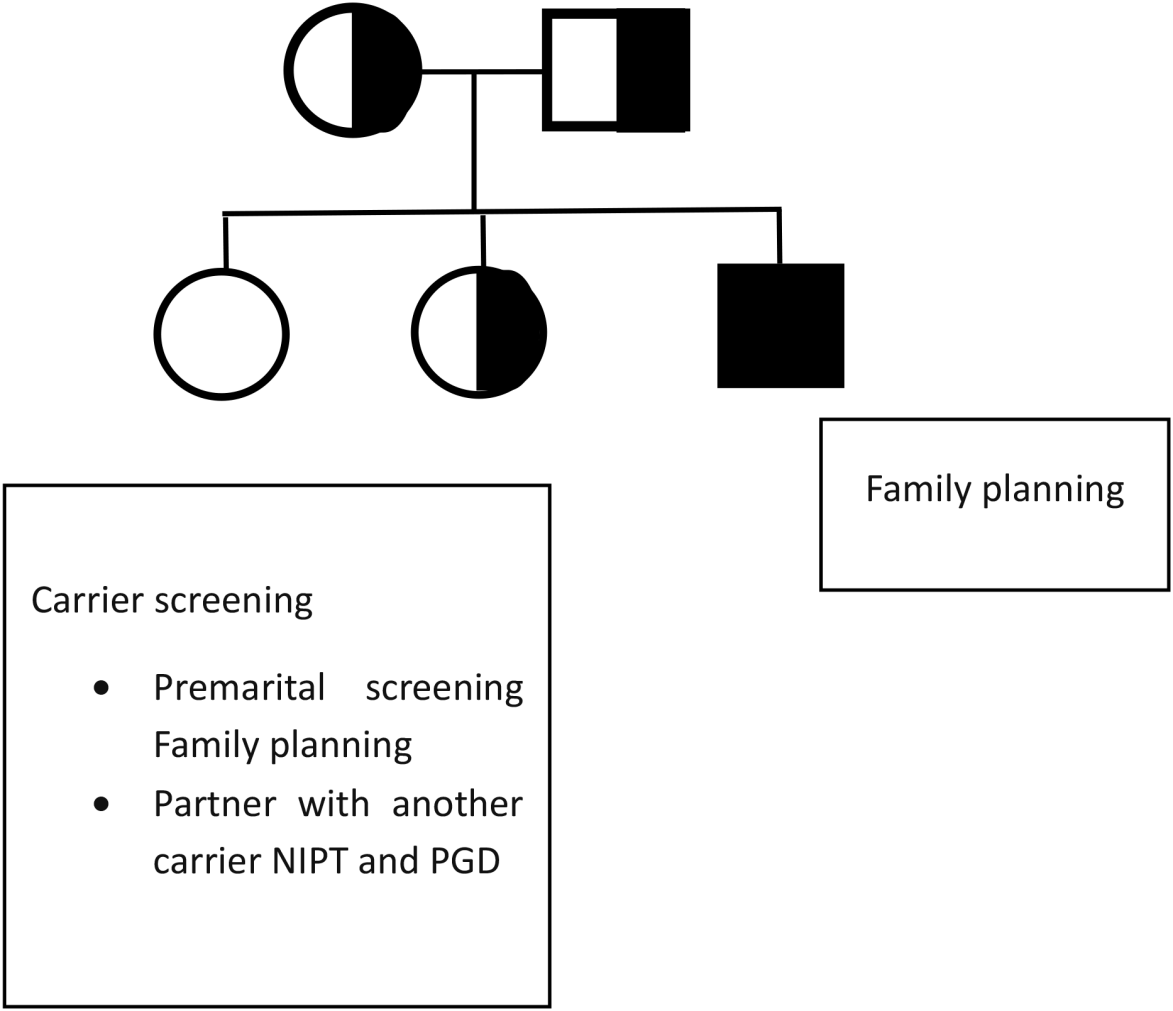
Family pedigree for an affected family, providing the preventive action for affected and carriers.

The first step in the screening procedure involves using family history to identify individuals at risk. Blood or saliva samples will be used for genetic testing, and molecular diagnostics will be used to check for CYP1B1 mutations (80). For those who test positive, genetic counseling will offer a comprehensive explanation of the risk, inheritance patterns, and reproductive alternatives for families willing to conceive or test recommendations for the pregnant mother (81). Carrier screening for other family members is valuable for understanding their future risk of passing the disease if they marry another carrier, as shown in **Figure 2**. Such screening procedures highlight the importance of targeted premarital testing for inherited familial conditions (1, 43).

### 5.3 Integration of AI tools and machine learning

Recent advancements in AI and machine learning have the potential to revolutionize genetic screening and early diagnosis of IODs:

#### 5.3.1 AI Applications in ocular diagnostics

Image Analysis: Algorithms are being developed to analyze OCT and fundus images with high precision, enabling clinicians to identify early signs of retinal degeneration with greater accuracy.

Predictive Modeling: Machine learning models can process complex genetic data alongside clinical features to predict disease progression and risk in individuals.

#### 5.3.2 Case studies and comparative analysis

In a study by Murro et al. (2023), a multidisciplinary team integrated next-generation sequencing (NGS) with advanced machine learning algorithms to improve the diagnosis and management of inherited retinal dystrophies. In this approach, patients suspected of having IRDs underwent both genetic testing and high-resolution retinal imaging. An AI model was then trained on the combined dataset to identify genotype–phenotype correlations, facilitating early and accurate diagnosis. Although the study focuses on IRDs, similar strategies may be adapted for other inherited ocular diseases such as congenital glaucoma or retinitis pigmentosa (72, 82).

#### 5.3.3 Proposed AI-enhanced screening pipeline

AI-assisted tools accomplish a high diagnostic accuracy (sensitivity/specificity) and support personalized care, through screening for prevention and early intervention.

##### 5.3.3.1 Data collection

**Figure 3:**
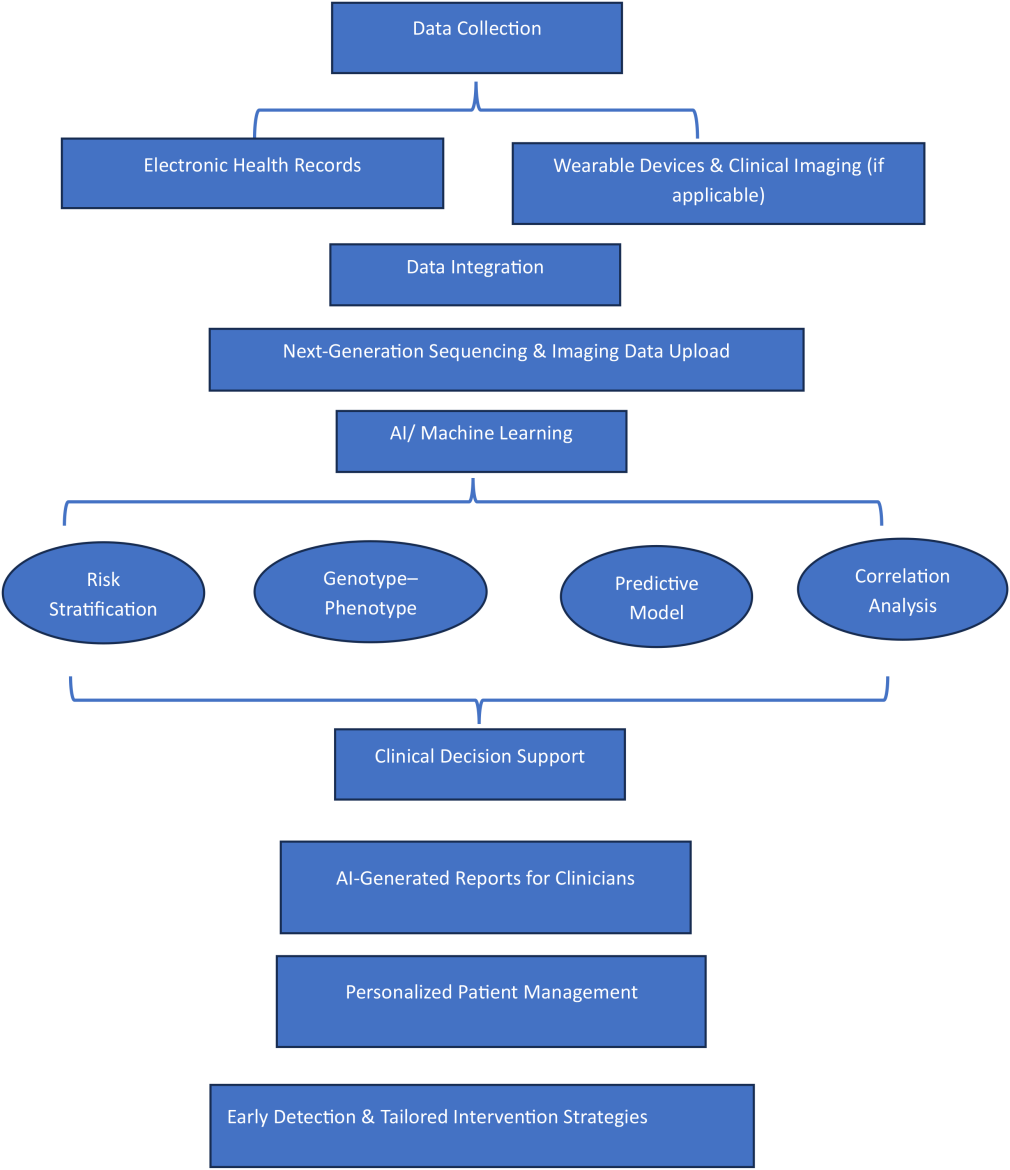
AI integration pipeline diagram. This diagram illustrates the data flow and decision-making in an AI-enhanced genetic screening program, demonstrating how data is collected, processed, and used to support personalized patient management.

Electronic health records EHRs) and wearable devices collect real-time patient data, as illustrated in **Figure 3**.

##### 5.3.3.2 AI processing

Machine learning algorithms analyze imaging and genetic data in real time, generating risk scores.

##### 5.3.3.3 Decision support

Clinicians receive AI-generated reports that aid in the diagnosis and management of IODs. With the advancement of AI transforming ophthalmology to detect retinal disease through OCT-based deep learning (CNN) classification (83), phenotype clustering in glaucoma (84), and diabetic retinopathy screening (84). ML methods like multimodal fusion, CNNs, and random forests enhance the detection of IRDs (85) and keratoconus (86).

##### 5.3.3.4 Remote clinic

For screening and follow-up, tele-ophthalmology platforms for remote screening (87). Providing equitable access to a broad spectrum of services across diverse populations in different regions.

## 6 Recommendations

Expanding screening services and adding more diseases to NBS programs in Saudi Arabia will improve early diagnosis and treatment, thereby lessening the nation’s long-term health and financial burden. Value-based healthcare aligns with a more comprehensive strategy, which enhances both sustainability and outcomes (41). Familial screening and a national registry for genetic ailments may enable early intervention and gene therapy, which can help prevent or postpone the onset or reduce the severity of rare AR diseases (47). The large proportion of consanguineous marriages highlights the challenge of changing marital norms, which requires a public health policy to activate PCG screening with a counselling program that facilitates cascade testing of silent carriers, and provides culturally sensitive support for consanguineous communities, providing family-planning decisions (52, 62). More government efforts will be needed to promote alternative reproductive options like pre-implantation genetic testing for monogenic diseases (PGT-M), and non-invasive prenatal testing (NIPT) (1, 62, 83).

## 7 Outcomes and expected impact

The main goal of the genetic screening program is to reduce the number of new IODs patients. Established as a preventive measure, this program aims to prevent PCG from being passed down to future generations by identifying carriers of the *CYP1B*1 mutation and offering genetic counseling. According to studies, genetic screening for early detection can significantly lower the incidence of illness, particularly in groups with high CM rates (88, 89).

## 8 Conclusion

Vision 2030 emphasizes preventive healthcare in Saudi Arabia; IODs pose significant challenges and a high prevalence of autosomal recessive disorders. Early detection through genetic screening offers substantial potential to reduce the disease burden. With the advancement of genetic testing, a tailored and personalized national screening program should be activated and integrated with genetic counseling, supporting families and alleviating the long-term healthcare burden. The program should also leverage technological advancements by incorporating telemedicine and AI-based predictive tools into the screening pipeline. The future incorporation of IODs into existing screening programs for early detection, prevention, family planning, and personalized medicine will lead to better outcomes on the national level.

## References

[1] Al Eissa MM Almsned F Alkharji RR Aldossary YM Alqurashi R . The perception of genetic diseases and premarital screening tests in the central region of Saudi Arabia. BMC Public Health. (2024) 24:1–15. doi: 10.1186/s12889-024-19029-0, PMID: 38858722 PMC11165879

[2] Eissa M Aloraini T Alsubaie L Alswaid A Eyiad W Mutairi F . Genetic carrier screening for disorders included in newborn screening in the Saudi population. J Biochem Clin Genet. (2021) 4:70–5. doi: 10.24911/JBCGenetics/183-1614266028

[3] WHO . International health regulations (2023). Available online at: https://www.who.int/health-topics/international-health-regulations.

[4] ALqahtani AS Alotibi R Aloraini T Almsned F AlAssali Y Ahmed A . Prospect of genetic disorders in Saudi Arabia. Front Genet. (2023) 14:1243518. doi: 10.3389/fgene.2023.1243518, PMID: 37799141 PMC10548463

[5] Badawi AH Al-Muhaylib AA Al Owaifeer AM Al-Essa RS Al-Shahwan SA . Primary congenital glaucoma: An updated review. Saudi J Ophthalmol. (2019) 33:382–8. doi: 10.1016/j.sjopt.2019.10.002, PMID: 31920449 PMC6950954

[6] Méjécase C Malka S Guan Z Slater A Arno G Moosajee M . Practical guide to genetic screening for inherited eye diseases. Ther Adv Ophthalmol. (2020) 12:1–28. doi: 10.1177/2515841420954592, PMID: 33015543 PMC7513416

[7] Reddy PCT Dandu R . Genetic testing for early detection and prevention of hereditary disorders. EPRA Int J Res Dev ( IJRD ). (2024) 7838:108–15.

[8] Vaishali K Surawanshi K . An investigative study into predictive modeling for early detection of eye disorders: challenges, strategies and mitigations. Int Res J Adv Eng Manag. (2024) 6:2448–56.

[9] Brar AS Parameswarappa DC Takkar B Narayanan R Jalali S Mandal S . Gene therapy for inherited retinal diseases: from laboratory bench to patient bedside and beyond. Ophthalmol Ther. (2024) 13:21–50. doi: 10.1007/s40123-023-00862-2, PMID: 38113023 PMC10776519

[10] Kristoffersson U Johansson-Soller M . Pregnancy planning and genetic testing: exploring advantages, and challenges. Genes (Basel). (2024) 15:1205. doi: 10.3390/genes15091205, PMID: 39336796 PMC11431595

[11] Talantova OE Postnikova TB Mikhailova AA Bespalova ON . Modern view of preconception carrier screening. J Obstet women’s Dis. (2024) 73:149–56. doi: 10.17816/JOWD623670

[12] Setiawan H Suhanda S . Genetic inheritance risk calculation as a practical approach in health prevention and management: A perspective. Genius J. (2024) 5:1–5. doi: 10.56359/gj.v5i1.341

[13] Jaffal L Joumaa H Noureldine J Banjak M Ibrahim M Mrad Z . The genetic landscape of inherited retinal dystrophies in Arabs. BMC Med Genomics. (2023) 16:1–9. doi: 10.1186/s12920-023-01518-7, PMID: 37127645 PMC10150479

[14] Alkuraya FS . Genetics and genomic medicine in saudi arabia. Mol Genet Genomic Med. (2014) 2:369–78. doi: 10.1002/mgg3.2014.2.issue-5 PMC4190871 25333061

[15] Milibari D Magliyah M Semidey VA Schatz P Albalawi HB . Unilateral retinitis pigmentosa associated with possible ciliopathy and a novel mutation. Clin Pract. (2022) 12:491–500. doi: 10.3390/clinpract12040053, PMID: 35892439 PMC9326729

[16] Aleissa M Aloraini T Alsubaie LF Hassoun M Abdulrahman G Swaid A . Common disease-associated gene variants in a Saudi Arabian population. Ann Saudi Med. (2022) 42:29–35. doi: 10.5144/0256-4947.2022.29, PMID: 35112591 PMC8812157

[17] Del Pozo-Valero M Almoallem B Dueñas Rey A Mahieu Q Van Heetvelde M Jeddawi L . Autozygome-guided exome-first study in a consanguineous cohort with early-onset retinal disease uncovers an isolated RIMS2 phenotype and a retina-enriched RIMS2 isoform. Clin Genet. (2024) 106:127–39. doi: 10.1111/cge.14517, PMID: 38468396

[18] Pandova MG Abduljalil T Elshafey AE Abdelmoaty SMA Albastawisy HI Bastaki LA . Inherited retinal dystrophies in a Kuwaiti tribe. Ophthalmic Genet. (2022) 43:438–45. doi: 10.1080/13816810.2022.2045509, PMID: 35272565

[19] Aldahmesh MA Safieh LA Alkuraya H Al-Rajhi A Shamseldin H Hashem M . Molecular characterization of retinitis pigmentosa in Saudi Arabia. Mol Vis. (2009) 15::2464–9. PMC2786884 19956407

[20] El-Ashry MF . Molecular genetics of corneal dystrophy. University College London (United Kingdom: University of London (2001).

[21] Molla A Jannadi R Alayoubi A Domlo H Alharbi Y Alrehaili Y . Impact of consanguinity and familial aggregation on vitiligo epidemiology in Saudi Arabia: a Case-control study. Cureus. (2024) 16:e63971. doi: 10.7759/cureus.63971, PMID: 39105022 PMC11299633

[22] Alsaif HS Khan AO Patel N Alkuraya H Hashem M Abdulwahab F . Congenital glaucoma and CYP1B1: an old story revisited. Hum Genet. (2019) 138:1043–9. doi: 10.1007/s00439-018-1878-z, PMID: 29556725

[23] Abu-Amero KK Sultan T Al-Obeidan SA Kondkar AA . Analysis of CYP1B1 sequence alterations in patients with primary open-angle glaucoma of Saudi origin. Clin Ophthalmol. (2018) 15:1413–6. doi: 10.2147/OPTH.S169943, PMID: 30127590 PMC6089601

[24] Ava S Demirtaş AA Karahan M Erdem S Oral D Keklikçi U . Genetic analysis of patients with primary congenital glaucoma. Int Ophthalmol. (2021) 41:2565–74. doi: 10.1007/s10792-021-01815-z, PMID: 33745036

[25] Badeeb OM Micheal S Koenekoop RK den Hollander AI Hedrawi MT . CYP1B1 mutations in patients with primary congenital glaucoma from Saudi Arabia. BMC Med Genet. (2014) 15:1–5. doi: 10.1186/s12881-014-0109-2, PMID: 25261878 PMC4258803

[26] Malik R Khandekar R Boodhna T Rahbeeni Z Al Towerki AE Edward DP . Eradicating primary congenital glaucoma from Saudi Arabia: The case for a national screening program. Saudi J Ophthalmol. (2017) 31:247–9. doi: 10.1016/j.sjopt.2017.08.002, PMID: 29234227 PMC5717496

[27] Ferreira JC Alshamali F Pereira L Fernandes V . Characterization of Arabian Peninsula whole exomes: exploring high inbreeding features. bioRxiv. 2022:2002–22. doi: 10.1101/2022.02.22.481461 PMC9619305 36325056

[28] Al-Towerki AE Gonnah ES Al-Rajhi A Wagoner MD . Changing indications for corneal transplantation at the King Khaled Eye Specialist Hospital (1983–2002). Cornea. (2004) 23:584–8., PMID: 15256997 10.1097/01.ico.0000121708.58571.5b

[29] Assiri AA Yousuf BI Quantock AJ Murphy PJ . Incidence and severity of keratoconus in Asir province, Saudi Arabia. Br J Ophthalmol. (2005) 89:1403–6. doi: 10.1136/bjo.2005.074955, PMID: 16234439 PMC1772915

[30] Reidy JJ . 2011–2012 basic and clinical science course–section 8: External disease and cornea. San Fr Am Acad Ophthalmol. (2011).

[31] Bialasiewicz A Edward DP . Corneal ectasias: study cohorts and epidemiology. Middle East Afr J Ophthalmol. (2013) 20:3–4. doi: 10.4103/0974-9233.106379, PMID: 23580846 PMC3617525

[32] Cheung IMY Angelo L Gokul A Ziaei M . Non-genetic risk factors for keratoconus and its progression. Clin Exp Optom. (2025), 1–9. doi: 10.1080/08164622.2024.2443454, PMID: 39762118

[33] Jahlan R Daghreeri A Otaif AA Otaif AA Hakami EF Mobarki AM . Public awareness of keratoconus and its association with eye rubbing behavior: A population-based study in Jazan, Saudi Arabia. Int J Med Dev Ctries. (2025) 9:1. doi: 10.24911/IJMDC.51-1737910935

[34] Ćwiek M Gimenez JB Izdebska J . Etiopathogenesis of keratoconus: A contemporary overview. Semin Ophthalmol. (2025) 12:1–13. doi: 10.1080/08820538.2025.2557985, PMID: 40937701

[35] Alzahrani AA Alnahdi AT Almehmadi MM Alzahrani EA . Eye rubbing: a survey of awareness of keratoconus and it’s relation with eye rubbing in Jeddah. (2021).

[36] Koenekoop RK Lopez I Den Hollander AI Allikmets R Cremers FPM . Genetic testing for retinal dystrophies and dysfunctions: benefits, dilemmas and solutions. Clin Experiment Ophthalmol. (2007) 35:473–85. doi: 10.1111/j.1442-9071.2007.01534.x, PMID: 17651254

[37] D’Andrea E Marzuillo C Pelone F De Vito C Villari P . Genetic testing and economic evaluations: a systematic review of the literature. Epidemiol Prev. (2015) 39:45–50., PMID: 26499415

[38] Ng QX Ong C Yaow CYL Chan HW Thumboo J Wang Y . Cost-of-illness studies of inherited retinal diseases: a systematic review. Orphanet J Rare Dis. (2024) 19:1–11. doi: 10.1186/s13023-024-03099-9, PMID: 38424595 PMC10905859

[39] Aleissa MM Alhawsawi AA Milibari D Schatz P Albalawi HB Alali NM . Genetics and clinical findings associated with early-onset myopia and retinal detachment in Saudi Arabia. (2025), 1–13. doi: 10.3390/genes16070848, PMID: 40725504 PMC12294696

[40] Ekram S Al Eissa MM . A comprehensive framework for the management of hereditary breast cancers : guiding light in precision medicine. (2025). doi: 10.3389/or.2025.1633387, PMID: 41049353 PMC12492225

[41] Alfadhel M Alhashem A Kurdi W Tulbah M Algamdi S Almannai M . Non-invasive prenatal testing in the kingdom of Saudi Arabia : current status of adoption and roadmap for the future non-invasive prenatal testing in the kingdom of Saudi Arabia : current status of adoption and roadmap for the future. (2025). doi: 10.2147/TACG.S535206, PMID: 41180264 PMC12577452

[42] Ashjan Alheggi A. A. M. T. E. Alhashem A Alshihry HMH Al-khenaizan S Alshammrie FF AlEissa MM . The saudi national policy and protocol for epidermolysis bullosa. (2025), 3521–3538, 2025. doi: 10.2147/RMHP.S532321, PMID: 41210910 PMC12593806

[43] Gene Vision . A resource on rare genetic eye disorders for everyone . Available online at: https://gene.vision/ (Accessed July 03, 2025).

[44] Saudi Ministry of Health . Premarital screening . Available online at: https://www.moh.gov.sa/en/HealthAwareness/EducationalContent/PublicHealth/Pages/PremaritalScreening.aspx (Accessed March 12, 2025).

[45] Alhusseini N Almuhanna Y Alabduljabbar L Alamri S Altayeb M Askar G . International newborn screening : where are we in Saudi Arabia. J Epidemiol Glob Health. (2024). doi: 10.1007/s44197-024-00263-z, PMID: 38922570 PMC11442708

[46] Lee YJ Ha A Kang D Shim SR Jeoung JW Park KH . Comparative efficacies of 13 surgical interventions for primary congenital glaucoma in children: a network meta-analysis of randomized clinical trials. Int J Surg. (2023) 109:953–62. doi: 10.1097/JS9.0000000000000283, PMID: 36999777 PMC10389407

[47] Abuzenadah A Alganmi N Alqurashi R Hawsa E Alotibi A . familial screening for the prevention of rare diseases : A focus on lipodystrophy in Southern Saudi Arabia. J Epidemiol Glob Health. (2024) 0123456789:162–8. doi: 10.1007/s44197-023-00182-5, PMID: 38231342 PMC11043304

[48] Grünauer-Kloevekorn C Winges A Stoye M Waibel A Biskup S Hoffmann K . Genetic counselling and gene analysis in patients with hereditary ocular diseases. (2023) 3:236–45. doi: 10.54352/dozv.BVVN4332

[49] Alizary A Ahmad K Al Bakri A . Parental experience with an ocular genetic counseling services in Saudi Arabia. Saudi J Ophthalmol. (2023). doi: 10.4103/sjopt.sjopt_154_23, PMID: 38155671 PMC10752285

[50] Haddad A Ait Boujmia OK El Maaloum L Dehbi H . Analysis of CYP1B1 gene mutations in primary congenital glaucoma patients. Eur J Ophthalmol. (2021) 31:2796–807. doi: 10.1177/11206721211016308, PMID: 34020567

[51] Bejjani BA Lewis RA Tomey KF Anderson KL Dueker DK Jabak M . Mutations in CYP1B1, the gene for cytochrome P4501B1, are the predominant cause of primary congenital glaucoma in Saudi Arabia. Am J Hum Genet. (1998) 62:325–33. doi: 10.1086/301725, PMID: 9463332 PMC1376900

[52] Alattas M Gordon S Sabin LL El-Jardali F Wirtz VJ . Equity and unmet need of non-communicable diseases services in Saudi Arabia using a National Household Survey (2019). BMC Health Serv Res. (2024) 24:346. doi: 10.1186/s12913-024-10787-6, PMID: 38491481 PMC10943914

[53] Ezzat H . PB2519: The effect of preventive health program on knowledge and screening of sickle cell disease among high school aged group–the saudi arabia experience. HemaSphere. (2023) 7:e8831295. doi: 10.1097/01.HS9.0000976776.88312.95

[54] Li J Chen X Yan Y Yao K . Molecular genetics of congenital cataracts. Exp Eye Res. (2020) 191:107872. doi: 10.1016/j.exer.2019.107872, PMID: 31770519

[55] Alasiri AA Mohammed V . Healthcare Transformation in Saudi Arabia: An Overview Since the Launch of Vision 2030. Heal Serv Insights. (2022) 15:1–7. doi: 10.1177/11786329221121214, PMID: 36081830 PMC9445529

[56] Kingdom of Saudi Arabia Vision 2030 . National transformation program delivery plan 2018-2020 . Available online at: https://vision2030.gov.sa/sites/default/files/attachments/NTP English Public Document_2810.pdf (Accessed March 24, 2025).

[57] Fidan ÇChecktae Akdur R Ünver ÇN Şahin ÖC Alper AB Ayhan A . Carrier screening programs for rare diseases in developed countries and the case of Turkey: A systematic review. Intractable Rare Dis Res. (2023) 12:161–9. doi: 10.5582/irdr.2023.01005, PMID: 37662625 PMC10468408

[58] Aljulifi MZ Almutairi MAS Ahmad MS Abdall SM Alelaiwi MMM Almutairi FLM . Awareness and acceptance of premarital screening test and genetic counseling program in Riyadh area, Saudi Arabia. Pakistan J Med Heal Sci. (2022) 16:875. doi: 10.53350/pjmhs22162875

[59] Hamamy H . Consanguineous marriages: Preconception consultation in primary health care settings. J Community Genet. (2012) 3:185–92. doi: 10.1007/s12687-011-0072-y, PMID: 22109912 PMC3419292

[60] MOH-Premarital Screening Program . Premarital screening . Available online at: https://www.moh.gov.sa/en/HealthAwareness/Beforemarriage/Pages/default.aspx (Accessed June 21, 2025).

[61] Parker LS . Ethical considerations in precision medicine. In: New Era Precis Med. Amsterdam, Netherlands: Elsevier (2024) p:143–72. doi: 10.1016/B978-0-443-13963-5.00002-9

[62] Rodríguez-Yunta E . Ethical and social issues in research on genetics and mental health. Salud Ment. (2023) 46:251–9. doi: 10.17711/SM.0185-3325.2023.032

[63] Smit AK Reyes-Marcelino G Keogh L Cust AE Newson AJ . [amp]]lsquo;There is a lot of good in knowing, but there is also a lot of downs’: public views on ethical considerations in population genomic screening. J Med Ethics. (2021) 47:e28–8. doi: 10.1136/medethics-2019-105934, PMID: 32434901

[64] Siermann M Valcke O Vermeesch JR Raivio T Tšuiko O Borry P . Are we not going too far?“: Socio-ethical considerations of preimplantation genetic testing using polygenic risk scores according to healthcare professionals. Soc Sci Med. (2024) 343:116599. doi: 10.1016/j.socscimed.2024.116599, PMID: 38244362

[65] Waldman L . The ethical, legal and psychosocial challenges of genetic testing: implications for primary medical care. Mo Med. (2004) 101:117–20., PMID: 15119109

[66] MOH . National newborn screening program continues . Available online at: https://www.moh.gov.sa/en/Ministry/MediaCenter/News/Pages/News-2017-11-27-003.aspx (Accessed March 23, 2025).

[67] Vision 2030 Projects . Saudi genome program . Available online at: https://www.vision2030.gov.sa/en/explore/projects/the-saudi-genome-program (Accessed July 14, 2025).

[68] Stone EM . Genetic testing for inherited eye disease. Arch Ophthalmol. (2007) 125:205–12. doi: 10.1001/archopht.125.2.205, PMID: 17296896

[69] Hu ML Edwards TL O’Hare F Hickey DG Wang JH Liu Z . Gene therapy for inherited retinal diseases: progress and possibilities. Clin Exp Optom. (2021) 104:444–54. doi: 10.1080/08164622.2021.1880863, PMID: 33689657

[70] Britten-Jones AC Gocuk SA Goh KL Huq A Edwards TL Ayton LN . The diagnostic yield of next generation sequencing in inherited retinal diseases: a systematic review and meta-analysis. Am J Ophthalmol. (2023) 249:57–73. doi: 10.1016/j.ajo.2022.12.027, PMID: 36592879

[71] Patel A Hayward JD Tailor V Nyanhete R Ahlfors H Gabriel C . The oculome panel test: next-generation sequencing to diagnose a diverse range of genetic developmental eye disorders. Ophthalmology. (2019) 126:888–907. 30653986 10.1016/j.ophtha.2018.12.050

[72] Murro V Banfi S Testa F Iarossi G Falsini B Sodi A . A multidisciplinary approach to inherited retinal dystrophies from diagnosis to initial care: a narrative review with inputs from clinical practice. Orphanet J Rare Dis. (2023) 18:223. doi: 10.1186/s13023-023-02798-z, PMID: 37525225 PMC10388566

[73] Bhargava S Mason L Okeke C . The significance of screening family members in glaucoma: opportunities and challenges. J Glaucoma. (2024) 33:S40–4. doi: 10.1097/IJG.0000000000002400, PMID: 38619402

[74] Founti P Stuart K Nolan WP Khawaja AP Foster PJ . Screening Strategies and Methodologies. J Glaucoma. (2024) 33:S15–20. doi: 10.1097/IJG.0000000000002426, PMID: 39149948

[75] Haas S Nattermann J Hüneburg R . Personalisierte prävention und früherkennung am beispiel des lynch-syndroms. Die Onkol. (2023) 29:859–67. doi: 10.1007/s00761-023-01360-7

[76] Alabbas AYS Al Balabel IAS Al Hayek NY Albalabel KJS Al Alhareth HMH Alyami WA . Innovative approaches to strengthening preventative care in contemporary healthcare: A systematic review. J Ecohumanism. (2024) 3:586–96. doi: 10.62754/joe.v3i7.4227

[77] Seyhan AA Carini C . Are innovation and new technologies in precision medicine paving a new era in patients centric care? J Transl Med. (2019) 17:1–28., PMID: 30953518 10.1186/s12967-019-1864-9 PMC6451233

[78] Shetty HP Patil MS Speciality C Yadav S . The Role of AI and Machine Learning in Revolutionizing Prenatal Screening and Genetic Analysis. (2024). doi: 10.38124/IJISRT

[79] Plugmann P . Innovation and future technology scenarios in health care: ideas and studies. Innov Technol Mark Leadersh Invest Futur. (2020) 4:31–43. doi: 10.1007/978-3-030-41309-5_4

[80] Kaur R Gupta N . Principles of Genetic Counseling in Eye Diseases. In: Genet Ocular Dis. (2022) p:195–210. doi: 10.1007/978-981-16-4247-0_16

[81] Abu-Amero KK Osman EA Mousa A Wheeler J Whigham B Allingham RR . Screening of CYP1B1 and LTBP2 genes in Saudi families with primary congenital glaucoma: genotype-phenotype correlation. Mol Vis. (2011) 17:2911., PMID: 22128238 PMC3224840

[82] De Fauw J Ledsam JR Romera-Paredes B Nikolov S Tomasev N Blackwell S . Clinically applicable deep learning for diagnosis and referral in retinal disease. Nat Med. (2018) 24:1342–50. doi: 10.1038/s41591-018-0107-6, PMID: 30104768

[83] Daich Varela M Sen S De Guimaraes TAC Kabiri N Pontikos N Balaskas K . Artificial intelligence in retinal disease: clinical application, challenges, and future directions. Graefe’s Arch Clin Exp Ophthalmol. (2023) 261:3283–97. doi: 10.1007/s00417-023-06052-x, PMID: 37160501 PMC10169139

[84] Shi NN Li J Liu GH Cao MF . Artificial intelligence for the detection of glaucoma with SD-OCT images: a systematic review and Meta-analysis. Int J Ophthalmol. (2024) 17:408–19. doi: 10.18240/ijo.2024.03.02, PMID: 38721504 PMC11074164

[85] Parmar UPS Surico PL Singh RB Romano F Salati C Spadea L . Artificial intelligence (AI) for early diagnosis of retinal diseases. Medicina (B Aires). (2024) 60:527. doi: 10.3390/medicina60040527, PMID: 38674173 PMC11052176

[86] Afifah A Syafira F Afladhanti PM Dharmawidiarini D . Artificial intelligence as diagnostic modality for keratoconus: A systematic review and meta-analysis. J Taibah Univ Med Sci. (2024) 19:296–303. doi: 10.1016/j.jtumed.2023.12.007, PMID: 38283379 PMC10821587

[87] El-Badawi K Goodchild C Drukarch H . Teleophthalmology in retinal. A Compr Overv Telemed. (2024) 26:153.

[88] Jamalalail BA . Perceived genetic knowledge, attitudes towards genetic testing, and the impact of genetic testing on parents of children affected with genetic disorders in Saudi Arabia. King Abdulaziz University Jeddah (2019).

[89] AbdulAzeez S Al Qahtani NH Almandil NB Al-Amodi AM Aldakeel SA Ghanem NZ . Genetic disorder prenatal diagnosis and pregnancy termination practices among high consanguinity population, Saudi Arabia. Sci Rep. (2019) 9:1–8. doi: 10.1038/s41598-019-53655-8, PMID: 31754150 PMC6872573

[90] Fighting Blindness Canada . Landmark study shows burden of inherited retinal diseases is borne primarily by families . Available online at: https://www.fightingblindness.ca/news/landmark-study-shows-burden-of-inherited-retinal-diseases-is-borne-primarily-by-families/?utm_source=chatgpt.com (Accessed May 24, 2025).

[91] Gong J Cheung S Fasso-Opie A Galvin O Moniz LS Earle D . The impact of inherited retinal diseases in the united states of america (Us) and canada from a cost-of-illness perspective. Clin Ophthalmol. (2021) 15:2855–66. doi: 10.2147/OPTH.S313719, PMID: 34234408 PMC8257071

